# Person-Centered Associations between High- and Low-Risk Personality Profiles and Psychological Adjustment in University Students

**DOI:** 10.1155/2024/8810153

**Published:** 2024-03-26

**Authors:** Rocco Servidio, Maria Giuseppina Bartolo, Flaviana Tenuta, Anna Lisa Palermiti, Francesca Candreva, Carmela Ciccarelli, Angela Costabile, Linda S. Pagani, Francesco Craig

**Affiliations:** ^1^Department of Cultures, Education and Society (DICES), University of Calabria, 87036 Cosenza, Italy; ^2^Psychological Counseling Services, University of Calabria, 87036 Cosenza, Italy; ^3^Department of Economics Statistics and Finance, University of Calabria, 87036 Cosenza, Italy; ^4^School of Psycho-Education, University of Montreal, Montreal, QC, Canada; ^5^School Environment Research Group, University of Montreal, Montreal, QC, Canada; ^6^Sainte-Justine's Pediatric Hospital Research Center, University of Montreal, Montreal, QC, Canada; ^7^Scientific Institute IRCCS “E. Medea”, Unit for Severe Disabilities in Developmental Age and Young Adults, Developmental Neurology and Neurorehabilitation, 72100 Brindisi, Italy

## Abstract

Personality traits are considered potential risk or protective factors for learning and psychological adjustment. This is a concern in higher education settings, which comprise mostly youth in emerging adulthood. The purpose of this study is to apply a person-centered approach to identify personality profiles of university students based on their character traits and then evaluate whether some clusters predict differences in emotional distress and coping strategies. We conducted a cross-sectional web-based survey with 467 southern Italian undergraduate university students (*M* = 21.8, SD = 3.69). Students completed an anonymous online survey and self-report questionnaires measuring sociodemographic characteristics, personality traits (Personality Inventory for DSM-5), emotional distress (General Anxiety Disorders-7, Patient Health Questionnaire-9), and coping strategies (Brief-COPE). Two distinct clusters were identified, differing in relation to maladaptive personality traits. One was characterized by high maladaptive personality traits, comprising 45.6% of the sample population. This high-risk profile evidenced higher levels of negative affect, detachment, psychoticism, antagonism, and disinhibition. A second cluster, with low maladaptive personality traits, represented the remainder of the sample. Participants featuring high maladaptive personality traits reported lower functioning in terms of avoidant coping strategies in comparison to the second low-risk cluster. Generating profiles of latent traits, such as in cluster analysis, can enhance a more profound theoretical understanding of underlying patterns within personality traits. This can enable higher education settings to meet variations in student needs by adapting their support services and interventions. Students can be trained to use coping strategies more effectively and efficiently.

## 1. Introduction

In recent years, the increase in mental health problems among university students has garnered the attention in the academic community, institutions, labor market, and organizations working in the field of mental health and productivity [1, 2]. This coincides with global increases in prevalence and incidence of depression across populations, especially in emerging adulthood [3–5]. Most psychological disorders begin during adolescence; however, three-quarters of lifetime mental disorders emerge before the age of 25 years [6–8]. Higher education often co-occurs during this developmental period. University students frequently contend with a combination of factors associated with psychological well-being, autonomy, and financial and social skills that either increase or decrease their susceptibility to emotional distress [9]. This vulnerability stems from the pivotal transition from adolescence to adulthood, which is characterized by profound identity shifts and heightened societal expectations [10]. Past research has identified factors contributing to poor student mental health, which include perceived academic pressures, financial stressors, social isolation, absence of familiar social or emotional support networks, and adapting to a new environment [11, 12].

Several recent epidemiological studies reveal that university students often experience depressive and/or anxious symptoms and elevated perceived psychological stress [13, 14]. For example, a meta-analysis of 64 studies [15] involving 100,187 university students found that the prevalence of depressive symptoms was 33.6% (with a 95% confidence interval ranging from 29.3% to 37.8%) and 39.0% for anxious symptoms (with a 95% confidence interval ranging from 34.6% to 43.4%). A recent longitudinal cohort study of over 350,000 students between 2013 and 2021 found that more than 60% met the diagnostic criteria for one or more mental health disorders [1]. This represents a remarkable increase of nearly 50% compared to the documented levels from 2013. Ahmed et al. reported a weighted mean prevalence of 39.65% (with a 95% confidence interval ranging from 35.72% to 43.58%) for nonspecific anxiety in undergraduate university students [16]. In medical students, Rotenstein et al. observed that the average occurrence of depressive symptoms was 27.2%, with the prevalence of suicide-related thoughts at 11.1%. These percentages are notably elevated, surpassing the figures observed in the general population [17].

Considering the prevalence of anxious and depressive symptomatology, both of which frequently entail a reduction in social and academic functioning and overall quality of life, it becomes crucial to examine putative mechanisms that increase psychopathological risk. It has been suggested that personality traits can be identified as risk and/or protective factors for anxiety and depression [18, 19]. The diathesis-stress model emphasizes the importance of understanding both biological and environmental factors in the development of psychological disorders. Some individuals may be genetically more vulnerable to developing certain mental disorders than others [19, 20]. This vulnerability can manifest through specific personality traits, such as high levels of fear and a tendency to interpret people and events in distorted, negative ways [21]. Thus, personality acts as one of the key components in the model, contributing to individual vulnerability and influencing the stress response to environmental challenges.

The 5-Factor Model of Personality, which corresponds to Big Five OCEAN dimensions [22], and the DSM-5 Alternative Model of Personality Disorder [23] are commonly used as a basis for understanding personality traits [24–26]. On one hand, a study with 580 university students found that the Big Five dimensions of neuroticism and agreeableness correlated with psychological distress and adjustment, respectively [27]. This suggests that personality assessments can identify at-risk students eligible for intervention. On the other hand, in a study conducted by Biondi et al. [28], students exhibiting social avoidance, reduced pleasure, and heightened negative emotions reported an increase in pandemic-related stress, with the psychoticism subscale of PID-5 consistently predicting suboptimal responses to such difficulties. Nevertheless, the relationship between personality traits and psychological well-being is multidimensional. This presents clinical challenges when predicting the risk of developing psychopathology symptoms. Accordingly, past research finds that individuals reporting high neuroticism often employ maladaptive coping strategies, and these can eventually contribute to the onset of depression and anxiety symptomatology [29, 30]. Studying the mechanisms involved in this relationship would be crucial for gaining a more comprehensive understanding of mental health in emerging adults.

Current research mainly focuses on exploring how individual personality traits impact anxious and depressive disorders. Yet, a paradigm shift is needed for a comprehensive understanding of risk along the spectrum of symptomatology that emanates profiles. The cumulative risk model argues that the risk of symptoms of mental distress, like depression and anxiety, is not solely based on one isolated personality trait [27]. Rather, the interaction between different traits could significantly amplify psychopathological risks. However, there remain limited data on how various personality profiles predict specific anxiety or depression symptoms in university students. Such information is warranted to guide effective intervention.

One method for reaching the research objective would be to apply cluster analysis. This statistical technique groups similar data points or observations into naturally occurring probability profiles, based on defined criteria or characteristics. This technique facilitates the identification of patterns and relationships within datasets that feature numerous variables and participants with similar underlying traits. In research applications, identifying distinct personality profiles sheds light on the interplay between specific traits and their impact on probability of adjustment/maladjustment [31, 32].

To comprehensively investigate the interrelationships within constructs, we operationalized personality traits using two distinct approaches: (1) a domain-specific method (e.g., variable-centered) and (2) a person-centered approach (e.g., profiles generated by cluster analysis). Linear models can be misleading and may overlook how the strength of each factor offsets other scales in the reported observations. Person-centered techniques such as cluster analyses offer some advantages. First, they account for the combined and cumulative impact of negative affect, detachment, psychoticism, antagonism, and disinhibition. They also yield results that identify distinct profiles that can serve many purposes, including facilitating and designing preventive intervention strategies.

To our knowledge, no studies have employed person-centered approaches, such as cluster analyses, to recognize the potential existence of subpopulations within the university student population characterized by unique combinations of parameters, including personality traits, coping mechanisms, and emotional distress. The motivation of this research is to explore subpopulations that could be characterized by unique combinations of parameters to detect nuanced patterns and identify distinct profiles that contribute to a more comprehensive understanding of the diverse psychological dynamics among university students.

Thus, the specific objectives of this study are twofold: (1) to identify clusters of personality traits in university students and (2) to investigate associations between high- and low-risk profiles and emotional distress and coping strategies. We expected to find distinct personality profiles that characterize risk for psychopathology and associations between such profiles and psychological adjustment within the university student population.

## 2. Methods

### 2.1. Participants and Procedures

We conducted this IRB-approved (protocol number: 0066896–September 2022), cross-sectional web-based survey study with undergraduate students at the University of Calabria in Rende (Cosenza, Italy), following the principles outlined in the Declaration of Helsinki. Participant recruitment occurred between September and December 2022. They were invited to participate in an anonymous online survey via QR codes, email invitations, advertisements distributed through the academic courses, and on-campus advertising. The estimated time to complete questionnaires was approximately 15-20 minutes. Inclusion criteria for participation were being age 18 years or older and possessing the ability to understand and complete a self-report questionnaire. Exclusion criteria included students with a certified diagnosis of mental or physical conditions that could impact their ability to complete the questionnaire accurately. Participants were recruited from various academic disciplines, including humanities, technology, medicine, pharmacy, social services, and education sciences degree programs. The average age of the overall sample was 21.8 years (SD = 3.69; range: age range 18-50 years). Questionnaires that were not fully answered were excluded. Convenience response sampling was used to obtain data using Google Forms. To reduce the possibility of repeat responses in the questionnaires, each student can only access the survey using their university identification code (limit to 1 response option). Additionally, time constraints can be set for questionnaire completion. This helps discourage individuals from completing the survey multiple times and can be particularly effective for online surveys. We clearly communicated to participants that multiple submissions are not allowed, as this may compromise the integrity of the research. The combination of these measures can enhance effectiveness in preventing repeated responses to the questionnaire.

The final sample comprised 467 participants, with signed and informed consent. Participants did not receive any incentives for their involvement. Responses were used exclusively for statistical analysis, following the European Data Protection Regulation GDPR 679/2016 to ensure privacy protection.

### 2.2. Measures

#### 2.2.1. Predictor: Personality Traits

Personality traits were assessed using the Brief Form of PID-5 [33, 34], a 25-item measuring tool that evaluates individual personality traits in alignment with the DSM-5 criteria. This self-report questionnaire covers five broad personality domains: negative affect, detachment, antagonism, disinhibition, and psychoticism. These five broad categories represent maladaptive interpretations of the well-established and FFM-validated “Big Five” personality trait inventory. They also bear similarities to the maladaptive counterparts found in the Personality Psychopathology Five (PSY-5). Negative affect is characterized by frequent and intense negative emotional experiences. Detachment involves avoiding social and emotional interactions by withdrawing and experiencing anhedonia. Antagonism refers to behaviors that go against societal norms and a lack of empathy. Disinhibition pertains to seeking immediate gratification. Finally, impulsivity and psychoticism refer to peculiar and incongruous behavior, both in its form and content, respectively. Each domain comprises several facets that further describe specific personality traits. Participants rate their agreement with each statement on a 5-point Likert-type scale, ranging from 1 “not at all true of me” to 5 “very true of me.” Each trait domain consists of 5 items. Higher scores within a specific domain reflect more pronounced maladaptive personality traits. The PID-5 brief version demonstrated good reliability, with a Cronbach's *α* coefficient of 0.83.

#### 2.2.2. Outcomes: Emotional Distress and Coping Strategies

*(1) Anxious Symptoms*. The GAD-7 [35] comprises a 7-item self-report scale designed to screen significant symptomatology of generalized anxiety disorder. Respondents are asked to rate the frequency and severity of symptoms experienced over the past two weeks, such as excessive worry, difficulty relaxing, and irritability. Scores range from 0 to 21, with higher scores indicating more severe generalized anxiety symptoms. Commonly used cut-off scores categorize individuals into different anxiety levels: 0 to 4 (minimal anxiety), 5 to 9 (mild anxiety), 10 to 14 (moderate anxiety), and 15 to 21 (severe anxiety). Based on established research and clinical practice, cut-off ranges are widely employed in both clinical and research contexts. The GAD-7 is a reliable and valid tool for screening generalized anxiety disorder symptoms. In our study, the GAD-7 demonstrated good reliability, with a Cronbach's *α* coefficient of 0.84.

*(2) Depressive Symptoms*. The PHQ-9 [36] comprises a 9-item questionnaire that assesses depressive symptoms, including loss of interest, changes in appetite, and feelings of hopelessness. Responses to each question are rated on a 4-point Likert-type scale ranging from 0 “never” to 3 “almost every day,” with higher scores indicating more severe depression symptoms. Cut-off scores are used to identify depression, with scores of 10 and above suggesting significant depression. The levels of depression, as indicated by the PHQ-9, include mild (scores 5-9), moderate (scores 10-14), moderately severe (scores 15-19), and severe (scores 20-27). The PHQ-9 is a commonly employed tool in clinical settings and is considered reliable and valid in assessing severity of depressive presentation. In our study, the overall PHQ-9 scale demonstrated very good reliability, with a Cronbach's *α* coefficient of 0.83.

*(3) Coping Strategies*. The Brief-COPE [37] is a 28-item self-report questionnaire designed to measure effective and ineffective coping strategies in response to stressful life events. It assists in identifying an individual's coping styles in counseling settings. The scale distinguishes primary coping styles into problem-focused strategies (such as active coping, planning, and instrumental support), emotion-focused strategies (including emotional support, positive reframing, acceptance, religion, and humor), and avoidant coping (involving self-distraction, denial, substance use, behavioral disengagement, venting, and self-blame). Responses are rated on a 4-point Likert-type scale, ranging from 1 “I haven't been doing this at all” to 4 “I've been doing this a lot.” Total scores were calculated for each coping style by summing the relevant items from each scale. Our findings indicated satisfactory internal consistency, with a Cronbach's *α* coefficient of 0.77.

### 2.3. Data Analytic Procedures

Descriptive statistics, univariate normality (skewness and kurtosis), and bivariate correlational analysis (Pearson *r*) were conducted to examine variable distributions as well as the relationships among them. Before conducting *K*-means cluster analysis, the data were scaled. Cluster analyses were then computed, based on the five standardised personality traits, to evaluate the distribution of subthreshold pathological personality trait facets within the sample. Cluster analysis was conducted using RStudio, with the following R libraries: cluster [38], factoextra [39], and gridExtra [40]. We applied squared Euclidean distance as the divergence measure between cases. The method of iterative revisions of clustered centroids was chosen for classifying cases, with new cluster centers being estimated after all cases were assigned to a given cluster.

To determine the optimal number of personality profiles from cluster analysis, two different algorithms were employed for method comparison: (1) the elbow method, which visually tests the consistency of the optimum number of clusters based on the square of the distance between the cluster centroid and the sample points in each cluster, and (2) GAP statistics, a data mining algorithm developed by Tibshirani et al. [41] to identify the number of *K* clusters that best represent the dataset. After comparing the results for different *K* values, we identified the most satisfactory solution with two clusters (*K* = 2). The analysis showed a small within-cluster variability compared to the difference between clusters, and the cluster sizes were greater than 10% of the total sample size.

A one-way ANOVA analysis was then computed to confirm the significance of differences in personality traits among the identified clusters. Finally, we analyzed relationships between personality profiles and coping strategies using a binomial logistic regression analysis based on the two identified clusters. These subsequent analyses were computed with the support of Jamovi and Stata packages.

## 3. Results

**Figure 1:**
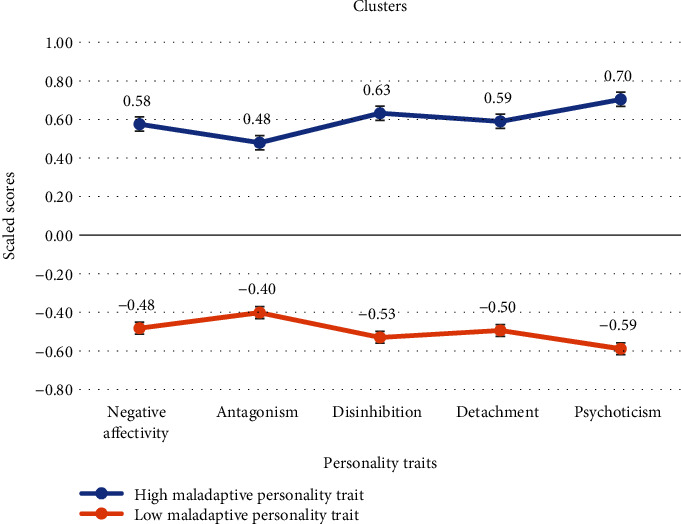
Mean personality profiles. High maladaptive behavior (cluster 1), with the highest mean values for all personality traits, comprises 45.6% of the sample, while cluster 2, representing 54.39%, exhibits lower mean values for the examined personality traits.

We reached out to a total of 600 southern Italian undergraduate university students for our survey, and we received responses from 467 of them (*M*_age_ = 21.8, SD = 3.69; 111 male and 356 female), resulting in an overall response rate of approximately 77.83%. The missing data were due to instances where participants either declined to participate or submitted incomplete questionnaires, leading to their exclusion from the study.  Table 1 reports descriptive statistics, reliability values, and relationships between variables using Pearson's bivariate correlations. Results from the *K*-means cluster analysis revealed two clusters. These were labelled to characterize personality traits in relation to psychological adjustment. The first was labelled high maladaptive personality traits. The second cluster was labelled low maladaptive personality traits. The mean values of both personality profiles are illustrated in Figure 1. As shown, high maladaptive behavior (cluster 1) comprised 45.6% (*n* = 213) of the sample and had the highest mean values for each personality trait. Conversely, cluster 2 included the low mean values of the personality traits and represented the 54.39% of the sample (*n* = 254).

For the current sample of university students, all the maladaptive personality traits were significantly higher in the first cluster. All score differences were significant according to the ANOVA analysis, as documented in Table 2, indicating that the two clusters differed.

A binary logistic regression was conducted to determine whether psychological distress and coping strategies could predict the two-cluster membership (high maladaptive personality trait vs. low maladaptive personality trait). The overall model was significant, *χ*2(5) = 135, *p* < 0.001, with 25.1% and 33.5% of the variance in the odds of low maladaptive behavior explained by the predictor set. Across both outcome categories, 71.9% of cases were accurately classified, with specificity (72.8%) being higher than sensitivity (70.9%). High maladaptive behavior was correctly predicted in 70.9% of cases compared to 72.8% of low maladaptive behavior.

The results of the binary logistic regression analysis (Table 3) suggest that university students with elevated levels of anxiety (OR = 1.083, 95% CI (1.109, 1.152)) and depression symptoms (OR = 1.213, 95% CI (1.140, 1.291)) are significantly more likely to be associated with the maladaptive personality profiles. Additionally, a significant association was observed between avoidant coping strategies (OR = 1.219, 95% CI (1.118, 1.329)) and the high maladaptive personality profile. No significant associations were found between the maladaptive personality profile and problem-focused coping or emotion-focused coping, respectively. The results of the current study also revealed that after adjusting for the coping strategies, the odds ratios for anxiety and depression changed slightly indicating a stable association with the high maladaptive personality profile.

## 4. Discussion

Using a person-centered approach, we identified two typical, yet distinct, personality risk profiles and evaluated whether these predicted differences in terms of psychological distress and coping strategies. The profiles differed from each other according to theoretical risk for maladjustment.

Past research has reported other variations using cluster solutions. Specifically, two studies found a three-cluster solution in patients with borderline personality disorder and individuals with at-risk mental distress [31, 32]. A possible explanation for deriving multiple solutions might be that clinical populations often exhibit a higher degree of variability and instability in risk presentations compared to nonclinical populations. Individuals in clinical populations may manifest a wide range of presentations which often correspond to symptoms of personality disorder. Our study is on typically developing youth in emerging adulthood. As such, the solutions estimated align with other studies that have examined student and nonclinical populations [42, 43]. Conversely, in a typically developing population, differences may be less pronounced, which can go overlooked when attempting to assess risks of maladjustment and disorder.

One of the key findings from this investigation was the identification of a profile that differed from others in terms of psychopathology risk. This group, with high maladaptive personality traits, comprised almost half our sample. They reported higher levels of negative affect, detachment, psychoticism, antagonism, and disinhibition compared with the other less at-risk profile. The size and features of this highly maladaptive personality profile suggest the heterogeneous nature of emotional and behavioral dispositions within undergraduate student populations. Assuming that this is a typical population, this means that many students would benefit from preventive intervention from their first semester onward.

As a secondary finding, we probed for dissimilarities in the levels of depression and anxiety among the delineated profiles. We found important distinctions across all measured psychological parameters. Individuals with highly maladaptive profiles reported the most emotional distress. Such individuals may be more susceptible to experiencing enough functional impairment from psychopathology to achieve a psychiatric diagnosis. More specifically, such individuals are more likely to experience frequent symptoms of sadness (and disinterest) and fear (and worry) at clinical levels compared with their counterparts with a more adjusted disposition [44]. Current evidence suggests that negative affect, characterized by a heightened tendency to experience negative emotion and cognitions, predicts increased chances of psychological distress [45]. Psychoticism, a trait characterized by unusual and unconventional thinking patterns, is more prone to cognitive distortions and unconventional beliefs, which could contribute to their unique coping strategies and psychological outcomes [46]. Heightened antagonism and disinhibition could potentially lead to interpersonal conflicts, risk-taking, or impulsive behavior [47]. This further exacerbates psychological distress. Furthermore, detachment may indicate difficulties in forming and maintaining interpersonal relationships, which in turn might exacerbate feelings of isolation and loneliness [48].

Recent literature is increasingly focused on investigating personality and emotional pathologies, such as depressive and anxious symptomatology, in both clinical and nonclinical samples [49, 50]. Our findings contribute to the literature by connecting depression and anxiety symptomatology with dimensional personality assessment. Within this context, recent evidence suggests that emotion dysregulation might serve as a transdiagnostic factor or risk marker across different disorders, including personality-related issues. Emotion regulation is a concept that can be observed to some degree and assessed either by clinical interview and neuropsychological evaluation. It plays a role in shaping both healthy and dysfunctional aspects of personality functioning. Stanton et al. discovered that while there is a significant intersection between personality dimensions and emotion regulation, both constructs independently make substantial contributions to predicting psychopathology. This underscores their potential significance in influencing mental health risks [51]. One recent study indicates that specific personality traits and emotion regulation styles play a significant role in predicting personality functioning among clinical samples of patients with depression and anxiety [52]. Our findings align with the notion that more vulnerable personality traits can forecast psychopathology, with emotional dysregulation as a common thread that underlies various maladaptive traits.

In addition, our findings highlight not only certain traits related to psychological adjustment but also the profile of personality traits associated with risk. It is plausible that the high maladaptive personality trait profile acts as a key amplifier of depression and anxiety. Theoretically, the AMPD provides a more nuanced and insightful analysis of personality traits, offering a key advantage for understanding and intervening with psychopathology. Moreover, when combined with person-centered approaches, it allows for the identification of at-risk groups within a university population. This individualized approach is particularly valuable, given the diverse landscape of cognitive and emotional challenges faced by university students. A person-centered approach for university populations can be valuable because risk of anxiety or depressive disorders is based on a dysfunctional personality profile. Consequently, clinical student services within campus settings, such as psychological counseling, can implement preventive measures and early interventions. The implementation of preventive measures and early interventions within these services emerges as a crucial strategy. This approach is pivotal not only in addressing the escalating demands placed on counseling services but also in response to the unique challenges presented by the post-COVID-19 era. In recent years, university counseling services have witnessed a notable surge in requests [53], partly attributed to shifts in teaching methodologies and a reduction in interpersonal interactions [54, 55]. The current study underscores the importance of considering personality traits when addressing mental health and well-being among students, highlighting that recognizing individual differences becomes crucial in tailoring counseling approaches and interventions effectively. Personalized strategies that align with students' unique personality traits could enhance the efficacy of mental health support, addressing not only immediate challenges but also fostering long-term resilience. Beyond identifying personality trait profiles, this study found that personality profiles differed in coping strategies. Those belonging to the high maladaptive personality profile reported being less functional in relation to the higher probability of selecting avoidant coping strategies. Avoidant coping refers to cognitive and behavioral actions aimed at reducing, negating, or disregarding the management of a stressful situation. Students with highly maladaptive traits may struggle because adopting avoidant coping strategies potentially unravels into other difficult situations, causing more stress. While some conceptualizations combine avoidant coping together with emotion-focused coping, it remains crucial to note that these styles have distinct functions and risks because of their use [56]. Avoidant coping primarily involves overlooking or ducking a stressor and is consequently passive in nature. Instead of mobilizing psychological resources (as in emotion-focused coping), avoidance can amplify the consequences of not meeting environmental demands. As such, our findings align with the scientific literature that highlights specific trends, as some studies have demonstrated a significant positive correlation between adaptive personality traits and active coping styles [29]. Personality characteristics associated with psychological instability and emotional dysregulation, such as those associated with neuroticism, predict avoidance coping [29]. The association between personality traits and coping styles implies that individuals with more vulnerable personalities are predisposed to experiencing psychological distress considering their typical deployment of maladaptive coping strategies like avoidance. Nevertheless, the findings concerning the relationship between personality and coping have not consistently yielded uniform results. Some researchers have failed to detect a significant connection between specific coping strategies and personality traits, such as agreeableness, conscientiousness, and openness [29].

Promising connections between personality and coping are slowly being established in the current literature on cognitive appraisal. Using path analysis with a large enough sample of undergraduate students, Chen et al. [57] found that personality predicts vulnerability to negative emotion (depression and anxiety) via two mediators: (1) the mindset pathway stress mindset (general belief about the nature of stress) and (2) coping flexibility (the ability to modulate away from ineffective coping strategies and choose alternative ones). They found that the stress-is-a-threat mindset mediated the association between stressful experiences and psychological vulnerability. Conversely, they found that the challenge–flexibility–enhancement (i.e., stress-is-a-challenge) mindset predicted coping flexibility toward lower levels of psychological distress, regardless of stressful experiences. Much like the findings reported here, neuroticism predicted the stress–threat–distress pathway mindset toward both psychological distress and cognitive inflexibility. Conscientiousness was associated with the challenge–flexibility–enhancement pathway, characterized by a stress-is-a-challenge mindset, which increased chances of coping flexibility toward less psychological distress. Extraversion, agreeableness, and openness were directly associated with greater coping flexibility which thus predicted less vulnerability. These relations speak directly to future directions in this research.

### 4.1. Future Directions

Our findings emphasize the importance of considering personality traits when addressing mental health and well-being among students. In terms of future implications, preventing academic challenges and mitigating maladjustment in university students likely reduces academic underachievement and dropout. Customized interventions and support mechanisms that promote more adaptive coping strategies and thus manage symptoms of depression and anxiety with identified groups of highly maladaptive personality profiles within student populations may prove effective and efficient in the prevention of disease and promotion of student well-being.

Indeed, there is a need for future research to delve deeper into the typical coping strategies used by individuals with these traits. This research could also explore methods to enhance their psychological resilience, providing a more comprehensive understanding of effective approaches to promote mental health in academic settings. Such insights are crucial for the design and implementation of targeted prevention strategies that cater to the unique needs of students, ultimately fostering a healthier and more successful academic journey while reducing the risk of academic attrition. Moreover, it is necessary to conduct more research and find ways to screen all university students, not just those who actively seek help. It remains important to consider that some students, who are either academically stressed or dysfunctional, might resist seeking professional help for their mental well-being as a typical avoidance strategy. This could lead to difficulties in handling the challenges of university life or emerging adulthood, resulting in lower academic performance and engagement, less support, and higher dropout rates. Institutional policies must remove existing organizational barriers to make university psychological counseling services available to all students, thus maximizing chances of academic retention and success. This would be promising news to supporters and contributors of publicly funded institutions of higher education.

### 4.2. Limitations

There are constraints associated with this study. First, a cross-sectional design does not prospectively forecast outcomes from one time to the next, while controlling for pre-existing and competing explanations. This limit is beyond questions of causality. Future research could adopt a longitudinal design, enabling multitime point examinations during university years. Second, while the sample size is comparable to that of prior studies involving university students, it may still be somewhat insufficient to capture all potentially significant distinctions within nonclinical populations. A larger sample could potentially capture a broader range of distinctions within nonclinical populations, revealing the potential existence of additional subpopulations within the university student population. Third, its reliance on self-report questionnaires and Internet-based data collection methods using online platforms are consistent with findings from traditional methods [29]. To address potential biases, researchers could use a mixed-methods approach, combining qualitative methods with online data. Exploring alternative in-person data collection methods could further validate results and enhance study robustness.

In conclusion, this study underscores the importance of a person-centered approach in predicting functional impairment risks based on individual characteristics, particularly among university students, as demonstrated in relevant studies [58, 59]. Our findings suggest that personality traits, such as negative affect, disinhibition, and psychoticism, or maladaptive coping strategies, could impair psychological adjustment and impact academic engagement at the university. Therefore, it offers universities a nuanced and individualized approach in formulating policy identification and promotion of student well-being and academic success.

## Figures and Tables

**Table 1:** Bivariate correlations (Pearson's *r*) and descriptive statistics for the main measures of the study.

(1) Anxiety	—	0.69⁣^∗∗∗^	0.56⁣^∗∗∗^	0.28⁣^∗∗∗^	0.40⁣^∗∗∗^	0.08	0.26⁣^∗∗∗^	0.44⁣^∗∗∗^	0.31⁣^∗∗∗^	0.07
(2) Depression		—	0.47⁣^∗∗∗^	0.45⁣^∗∗∗^	0.51⁣^∗∗∗^	0.13⁣^∗∗^	0.29⁣^∗∗∗^	0.52⁣^∗∗∗^	0.29⁣^∗∗∗^	-0.01
(3) Negative affectivity			—	0.30⁣^∗∗∗^	0.47⁣^∗∗∗^	0.11⁣^∗^	0.22⁣^∗∗∗^	0.40⁣^∗∗∗^	0.27⁣^∗∗∗^	0.01
(4) Detachment				—	0.41⁣^∗∗∗^	0.25⁣^∗∗∗^	0.31⁣^∗∗∗^	0.33⁣^∗∗∗^	0.01	-0.31⁣^∗∗∗^
(5) Psychoticism					—	0.30⁣^∗∗∗^	0.37⁣^∗∗∗^	0.46⁣^∗∗∗^	0.16⁣^∗∗∗^	0.00
(6) Antagonism						—	0.37⁣^∗∗∗^	0.25⁣^∗∗∗^	0.14⁣^∗∗^	0.06
(7) Disinhibition							—	0.29⁣^∗∗∗^	0.03	-0.08
(8) Avoidant coping								—	0.35⁣^∗∗∗^	0.08
(9) Emotion-focused coping									—	0.55⁣^∗∗∗^
(10) Problem-focused coping										—
Mean	11	9.92	8.54	5.16	5.64	2.71	4.59	14.92	28.01	20.45
SD	4.59	5.18	2.67	2.76	3.09	2.34	2.53	3.25	5.38	4.71
Skewness	0.13	0.70	-0.18	0.41	0.27	1.03	0.43	0.75	0.35	0.17
Kurtosis	-0.88	0.18	-0.28	-0.22	-0.34	0.86	0.07	0.93	0.03	-0.38

Note. ⁣^∗^*p* < 0.05; ⁣^∗∗^*p* < 0.01; ⁣^∗∗∗^*p* < 0.001.

**Table 2:** Comparison of personality trait scores among the two identified clusters (*N* = 467).

Personality traits	High maladaptive personality trait (mean (SD))	Low maladaptive personality trait (mean (SD))	*F* ratio	*p*	*η* ^2^
Negative affectivity	10.08 (2.17)	7.25 (2.35)	180	0.001	0.28
Detachment	6.79 (2.45)	3.80 (2.20)	193	0.001	0.29
Psychoticism	7.82 (2.50)	3.82 (2.25)	332	0.001	0.42
Antagonism	3.83 (2.60)	1.77 (1.57)	111	0.001	0.19
Disinhibition	6.19 (2.29)	3.25 (1.86)	235	0.001	0.34

**Table 3:** Binomial logistic regression results for the prediction of the two clusters as a function of the predictor variables.

	Exp(*B*)	95% CI for Exp(*B*)	*B*	SE	*t*	*p*
Block 1						
Constant	0.051	[0.027, 0.098]	-2.969	0.329	-9.03	0.001
Anxiety	1.083	[1.019, 1.152]	0.080	0.031	2.55	0.011
Depression	1.213	[1.140, 1.291]	0.193	0.032	6.11	0.001
Block 2						
Constant	0.004	[8.344, 0.021]	-5.473	0.824	-6.64	0.001
Anxiety	1.065	[0.998, 1.136]	0.063	0.033	1.91	0.056
Depression	1.156	[1.083, 1.235]	0.145	0.033	4.33	0.001
Problem coping	0.952	[0.898, 1.009]	-0.049	0.029	-1.64	0.101
Emotion coping	1.043	[0.989, 1.101]	0.043	0.027	1.56	0.120
Avoidant coping	1.219	[1.118, 1.329]	0.198	0.044	4.49	0.001

Note. CI = confidence interval. The reference category for the dependent variable was “cluster 2–low maladaptive personality trait.” Exp(*B*) = odds ratio.

## Data Availability

The data that support the findings of this study are available from the first or corresponding author upon request.
